# Small Molecule Inhibitors of Influenza Virus Entry

**DOI:** 10.3390/ph14060587

**Published:** 2021-06-18

**Authors:** Zhaoyu Chen, Qinghua Cui, Michael Caffrey, Lijun Rong, Ruikun Du

**Affiliations:** 1 College of Pharmacy, Shandong University of Traditional Chinese Medicine, Jinan 250355, China; chenzhaoyu666@163.com (Z.C.); cuiqinghua1122@163.com (Q.C.); 2 Experimental Center, Shandong University of Traditional Chinese Medicine, Jinan 250355, China; 3 Qingdao Academy of Chinese Medicinal Sciences, Shandong University of Traditional Chinese Medicine, Qingdao 266122, China; 4 Department of Biochemistry and Molecular Genetics, University of Illinois at Chicago, Chicago, IL 60607, USA; caffrey@uic.edu; 5 Department of Microbiology and Immunology, College of Medicine, University of Illinois at Chicago, Chicago, IL 60612, USA

**Keywords:** influenza virus, hemagglutinin, small molecule inhibitor, fusion machinery

## Abstract

Hemagglutinin (HA) plays a critical role during influenza virus receptor binding and subsequent membrane fusion process, thus HA has become a promising drug target. For the past several decades, we and other researchers have discovered a series of HA inhibitors mainly targeting its fusion machinery. In this review, we summarize the advances in HA-targeted development of small molecule inhibitors. Moreover, we discuss the structural basis and mode of action of these inhibitors, and speculate upon future directions toward more potent inhibitors of membrane fusion and potential anti-influenza drugs.

## 1. Introduction

Influenza viruses are enveloped viruses that belong to the family *Orthomyxoviridae*, and can be classified into four types: A, B, C and the recently identified type D [1], among which influenza A virus (IAV) as well as IBV and ICV infect humans. IAV and IBV are responsible for the seasonal epidemics, which cause up to 650,000 respiratory deaths worldwide annually [1]. In addition, four large scale IAV pandemics (Spanish flu in 1918, Asian flu in 1957, Hong Kong flu in 1968, and swine flu in 2009) have occurred historically, bringing tremendous loss of human lives [2]. Moreover, it is of great concern that antigenically novel zoonoticIAVs can occasionally cross the species barrier and infect humans with high rates of morbidity and mortality, posing potential pandemic risks [3,4].

Standard trivalent or quadrivalent recombinant influenza vaccines provide cost-effective protection against seasonal influenza, however, circulating IAVs readily evolve, which requires that the vaccine composition be reviewed each year to account for antigenicity changes, and the vaccine effectiveness varies from year to year with average protection rates of 50–60% [5]. Moreover, the protection conferred by seasonal influenza vaccines poorly covers emerging pandemic influenza virus strains [5].

Antiviral drugs are another major countermeasure to combat influenza virus infection. At present, there are three classes of antiviral drugs available against influenza virus infection, including the viral ion channel M2 blockers (amantadine and rimantadine), neuraminidase (NA) inhibitors (oseltamivir, zanamivir, and peramivir) and the polymerase inhibitors (favipiravir and baloxavir). However, M2 blockers are not recommended for clinical use any longer since 99% of the circulating influenza strains are resistant to them [6], while increasing evidence also showed that resistance to NA inhibitors impedes their efficacy [7]. Although baloxavirwas recently approved in the US and Japan [8,9], mutations responsible for reduced susceptibility of IAVs to baloxavir have been detected [10,11]. In addition, a universal mechanism for favipiravir resistance has been well illustrated [12,13].

Taken together, the limitations of current vaccines and antivirals underscore the importance of novel anti-influenza treatments. To date, the life cycle of influenza virus has been well understood, allowing for the validation of multiple intervention points [14]. Hemagglutinin (HA), which mediates the initial entry step of virus infection, is one of the most appealing drug targets [15]. In this review, we summarize the current development of small molecule HA inhibitors that block influenza virus entry.

## 2. The Structure and Function of HA

IAV and IBV encode two major surface glycoproteins, HA and NA. HA mediates receptor binding and membrane fusion during influenza virus entry, while NA mainly facilitates release of the newly budded virions from host cells [16]. However, ICV and IDV encode only one surface glycoprotein, the haemagglutinin-esterase-fusion (HEF) protein, which combines both the function of HA and NA [17,18]. Considering ICV usually causes mild infections while IDV does not infect humans, we will not discuss ICV and IDV in this review, and “influenza virus” thereafter refers to IAV and IBV to avoid misunderstanding.

**Figure 1:**
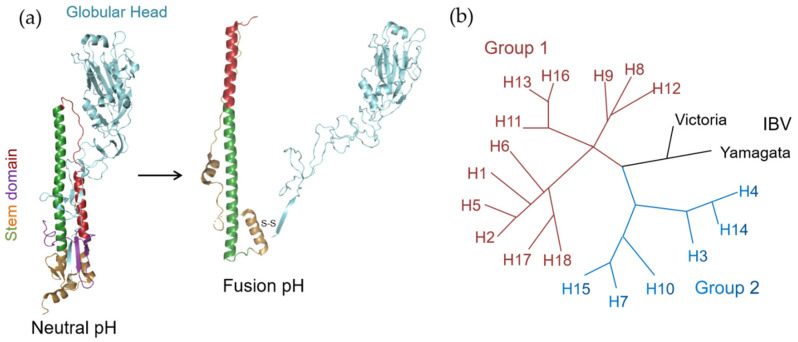
(**a**) The structure of a monomer of the HA trimer at neutral pH and the rearranged structure at fusion pH.The subunits HA1 and HA2, are colored blue and multicolored, respectively. The central helix was shown in green, while the short helix was shown in red. Note that the loop between central and short helixes undergoes a “loop-to-helix” transition when induced by lower pH. (**b**) Phylogenetic tree of influenza HAs. The two groups of IAV are colored brown (group 1) and blue (group 2), while IBV HAs are indicated in black.

HA is primarily translated as an HA0 precursor, and assembled as a homotrimer in the endoplasmic reticulum. During the virus maturation process, HA0 is further cleaved into an HA1–HA2 complex by host proteases. The mature HA1–HA2 complex consists of two domains: the membrane-distal globular head domain comprised of HA1, containing a receptor binding site, and the membrane-proximal helix-rich stem domain, primarily composed of HA2 with some HA1 residues, containing a fusion machinery (Figure 1a) [19]. Entry of the influenza virus is initiated when HA binds the receptor sialic acid (SA) on the host cell surface followed by endocytosis. The low pH condition of the maturing endosome then triggers a series of conformational changes of HA, including exposure of the hydrophobic fusion peptide and “loop-to-helix” transition of HA2, which ultimately result in the fusion between viral and host endosomal membranes (Figure 1a) [20].

A major hurdle that impedes the development of HA inhibitors is the high pleomorphicity of HA. To date, 18 antigenic subtypes (H1–H18) of IAV HA have been discovered. Based on phylogenetic analysis, these subtypes fall into two groups. Group 1 encompasses H1, H2, H5, H6, H8, H9, H11, H12, H13, H16, H17 and H18, while group 2 encompasses H3, H4, H7, H10, H14 and H15 (Figure 1b) [21]. In addition, there are two distinct classes of IBV HAs, Yamagata-like and Victoria-like lineages (Figure 1b) [11]. The HAs of current seasonal IAVs, H1 and H3, are members of groups 1 and 2, respectively, as are the highly pathogenic viruses of the H5 (group 1) and H7 (group 2) subtypes [1,4,22].

The head region of HA is highly plastic, however, the receptor binding site (RBS) is relatively conserved and has been recognized as an attractive drug target [23]. In contrast, the stem domain is the least variable region of HA, and numerous small molecules have been identified to target this region and block virus infectivity by inhibiting HA-mediated membrane fusion [21,24].

## 3. Small Molecule Inhibitors Targeting HA Mediated Receptor Binding

Discovery or design of small-molecule therapeutics that specifically target the receptor binding pocket has been challenging, for the SA-binding pocket of HA is small and shallow, and the monovalent binding affinity of HA for SA is low [25]. The strong virus-cell adhesion of influenza viruses depends on multivalent interactions of HA with densely distributed SAs at cell surface [26]. Therefore, the straightforward strategy of mimicking receptor SA by carbohydrate-based analogs to block virus-receptor binding is difficult to achieve, since monovalent SA derivatives could hardly compete with native glycans [27]. As an alternative, great effort has been focused on designing 3D scaffolds carrying multivalent SA analogs to inhibit influenza virus infection [28,29,30,31].

**Figure 2:**
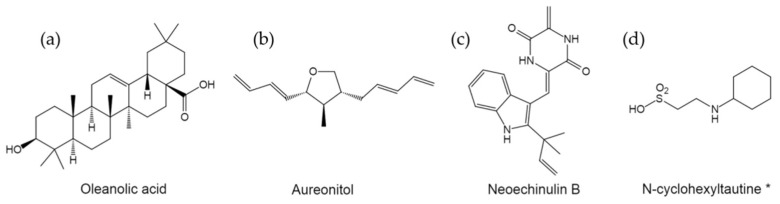
HA inhibitors targeting the receptor binding site. (**a**–**d**) The structures of oleanolic acid (**a**), aureonitol (**b**), neoechinulin B (**c**), and N-cyclohexyltautine (**d**). *, the inhibition has not been validated by bioassays.

Non-carbohydrate small molecule inhibitors targeting the RBS of influenza HAs have also been discovered. Previously, pentacyclic triterpenoids (PTs) have been demonstrated to possess broad-spectrum antiviral activities. The anti-influenza activity of PTs was therefore tested and it showed that PTs such as oleanolic acid (OA, Figure 2a) and ursolic acid can effectively inhibit IAV replication [32]. Mechanism of action (MOA) studies indicated that OA can inhibit HA-mediated hemagglutination, and docking studies suggest that the SA binding pocket within HA potentially acts as a target domain [32]. Moreover, OA showed a broad anti-influenza spectrum and a diminished tendency to induce drug resistance [32]. Subsequently, structure-activity relationship (SAR) studies were conducted using OA as lead compound, and a series of PT-derivatives exhibiting higher anti-influenza potency were developed [32,33,34,35,36,37], for example, Meng et al.developed an OA-arginine conjugate that exhibited robust potency and broad antiviral spectrum with EC_50_ values at the low-micromolar level [35].

In addition, a natural product derived from the fungus *Chaetomium* Kuntze ex Fries (*Chaetomiaceae*), aureonitol (Figure 2b), also shows effective inhibition against both IAV and IBV [38]. MOA studies demonstrated that aureonitol inhibits influenza hemagglutination and consequently impairs virus adsorption significantly [38]. Molecular modeling studies suggested that aureonitol can fit well to the SA binding site of HA and interact with several highly conserved residues via hydrogen bonds [38]. Moreover, Chen et al. identified neoechinulin B (Figure 2c), which is a prenylated indole diketopiperazine alkaloid derived from marine-derived fungus *Eurotium rubrum*, to exert potent inhibition against a panel of influenza virus strains, including amantadine- and oseltamivir-resistant clinical isolates [39]. MOA studies indicated that neoechinulin B can bind to influenza HA, and disrupt HA-receptor interaction and virus attachment to host cells [39]. Both aureonitol and neoechinulin B provide new leads for the development of potential influenza virus inhibitors targeting HA-mediated receptor binding.

Interestingly, Kadama and Wilson noted that a small molecule fragment, *N*-cyclohexyltaurine (Figure 2c), commonly known as the buffering agent CHES, can bind to HA emulating with SA and RBS-targeting broadly neutralizing antibodies [40]. The crystal structure of *N*-cyclohexyltaurine in complex with group 1 HA of H5N1 A/Vietnam/1203/2004 (H5/Viet) indicated that *N*-cyclohexyltaurine interacts with RBS by mimicking the binding mode of SA [40]. Moreover, for HA of H3N2A/Hong Kong/1/1968 (H3/HK68), *N*-cyclohexyltaurine also binds to a conserved pocket in the stem region in addition to the RBS, thereby exhibiting a dual-binding mode in group 2 HAs [40]. Note that the binding of *N*-cyclohexyltaurine to HA is non-specific and it has been reported that *N*-cyclohexyltaurine also binds to the catalytic domain of *Clostridium perfringens* neuraminidase [41]. Moreover, the anti-influenza potency of *N*-cyclohexyltaurine has not been validated by any bioassay. Nonetheless, the structural insights into RBS recognition by a small molecule can serve as a template to guide further development of novel small-molecule therapeutics against influenza virus.

## 4. Small Molecule Inhibitors Targeting HA Mediated Fusion

Numerous small molecule HA inhibitors targeting the fusion machinery have been developed, although most of these fusion inhibitors appear to operate in a group specific or even subtype specific manner. So far to our knowledge, none of the small molecule HA fusion inhibitors have entered into clinical trials, except Arbidol, which was approved and launched in Russia in 1992, although its activity is not limited to HA inhibition (the details will be discussed later). In the following subsections, we review the discovery and chemical optimizations of these influenza fusion inhibitors, and information about the specificity, in vitro and in vivo activities are summarized in Appendix A.

### 4.1. Group 1 Specific Influenza Fusion Inhibitors

#### 4.1.1. Benzenesulfonamides—The First Generation Orally Active HA Inhibitors

**Figure 3:**
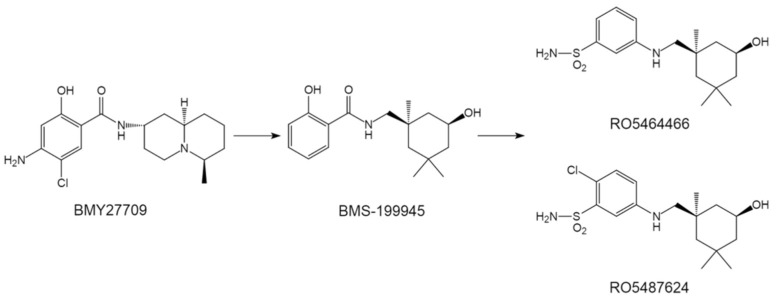
The structures of BMY27709 and its derivatives.

As early as 1996, Luo et al. identified an influenza inhibitor BMY27709 (Figure 3), that specifically targets group 1 HA fusion [42,43]. SAR studies associated with BMY27709 were subsequently examined using a parallel synthesis approach, and a new compound BMS-199945 (Figure 3) was synthesized and showed an increased inhibitory effect displaying EC_50_ values of 0.06–0.42 μmagainst group 1 IAVs [44,45]. Further, using BMS-199945 as starting point, Tang et al. further designed and synthesized a class of benzenesulfonamide derivatives as novel HA inhibitors, among which RO5464466 and its analogue RO5487624 showed comparable antiviral potency with compound BMS-199945 (Figure 3). However, pharmacokinetics study revealed that compared to compound BMS-199945, RO5487624 shows good oral availabilities, significantly improved in vivo stability and longer terminal half-life [46,47]. Further, RO5487624 displayed a significant protective efficacy on mice in terms of survival rate [47]. These benzenesulfonamides represent the first generation of orally bioavailable HA inhibitors that have potential for further development.

#### 4.1.2. JNJ4796—One of the Most Potent Drug Candidates

The development of JNJ4796 was inspired by the advances of universal influenza vaccines and broadly neutralizing antibodies (bnAbs) [48]. Previously, an HA stem-targeting bnAb CR6261, which broadly neutralizes most group 1 IAVs has been identified and the co-crystal structure of CR6261 in complex with H1 HA has been resolved [49,50]. These findings stimulated the design of small proteins that mimic the antibody interaction with HA and inhibit influenza virus fusion [46,51,52]. In addition, based on the co-crystal structures of bnAbs FI6v3 and CR9114 with HAs, smaller peptidic influenza fusion inhibitors also have been designed [53].

Small-molecule mimics of the bnAb CR6216 were next pursued, as small molecules possess advantages of oral bioavailability, high shelf stability, and relatively low production costs. However, it is much more challenging to develop small molecule inhibitors directed at antibody binding sites, since the antibody epitopes are protein-protein interfaces and are generally flat, large, and undulating, in contrast to small concave pockets of common targets for small molecule drugs [48]. Moreover, small molecule inhibitors mimicking the function of an HA-stem bnAb should reproduce the key interactions that lead to fusion inhibition [48].

**Figure 4:**
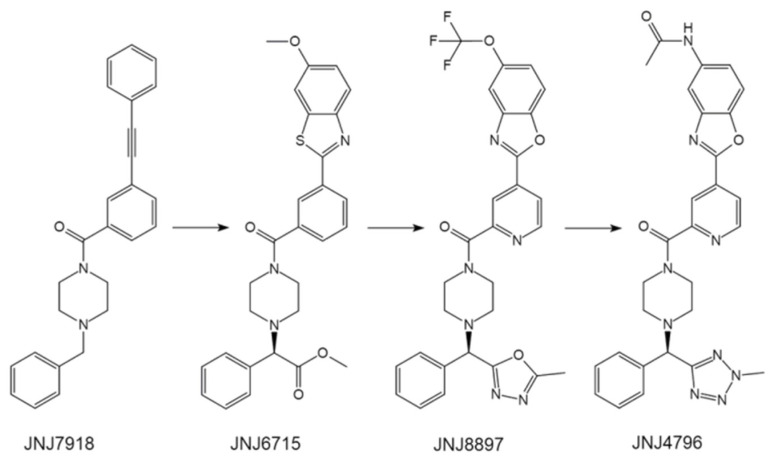
The structure of JNJ7918 and its chemical modification process. JNJ6715, key modifications were made to improve the HA stem-binding property. JNJ8897, key modifications were made to improve the drug-like property. JNJ4796, key modifications were made to improve the pharmacokinetics property.

In order to identify potent small molecules that mimic CR6216, Dongenet al. set out to utilize the structural basis of the CR6261-HA complex and established an amplified luminescent proximity homogeneous assay (AlphaLISA) in competition mode as a high-throughput screening (HTS) method. Encouragingly, the researchers identified benzylpiperazines from ~500,000 small molecule compounds as a major hit class, with JNJ7918 being the most promising lead compound to prevent the CR6261-HA interaction (Figure 4). Chemical modifications were subsequently introduced sequentially to improve the activity and properties dictating metabolic stability and oral bioavailability. Finally, an orally active small molecule fusion inhibitor of influenza virus, JNJ4796, was generated (Figure 4). Consistent with the breath of CR6261 binding, JNJ4796 binds and neutralizes a broad spectrum of group 1 IAVs.

Recently, increasing numbers of antibody epitopes that are conserved in both groups 1 and 2 HAs have been identified [54,55,56,57,58]. These novel epitopes may serve as attractive targets for the development of more valuable pan-subtype small molecule HA inhibitors, inspired by the discovery of JNJ4796.

#### 4.1.3. CBS1116—Variable Directions to Chemical Optimization

**Figure 5:**
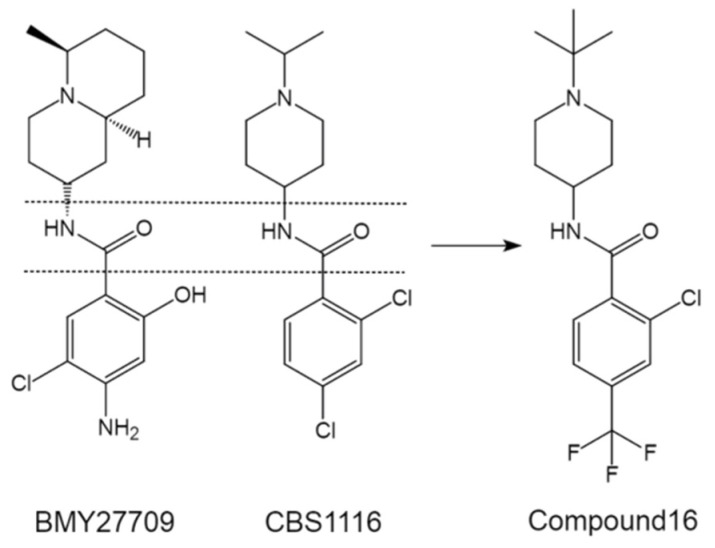
Comparison of CBS1116 with BMY27709, and the structure of optimized compound 16 derived from CBS1116.

During the past two decades, our group has engaged in the discovery of novel entry inhibitors of influenza viruses, including the high-pathogenic IAVs H5N1 and H7N3, mainly by using an optimized comparative HTS approach on the basis of pseudotyped viruses [59]. CBS1116 (2,4-dichloro-N-(1-isopropyl-4-piperidinyl) benzamide), from the commercially available Chembridge small molecule library (19,200 compounds), is one of the most promising hit compounds that show group 1 specific inhibitory effect against IAV entry [21]. MOA studies indicated that CBS1116 does not interfere with HA binding but acts against the HA-mediated fusion process. Interestingly, we noticed that CBS1116 share similar scaffold with BMY27709 (Figure 5). Both of the two compounds contain a critical benzene ring, an amido bond as linker, and a second variable ring, i.e., a piperidine for CBS1116 and a quinolizidine for BMY27709.

We subsequently focused on the unique 4-aminopiperidine moiety of CBS1116 and developed a comprehensive SAR, by modifying appropriate substituents in the amide portion of the molecule, the tertiary amine region as well as the aromatic region [60]. Encouragingly, we finally generated compound 16, which exhibits an in vitro EC_50_ of 72 ± 24 nm in a reporter IAV-based luciferase assay (Figure 5) [60]. In addition, we demonstrated that the combination of compound 16 with the NA inhibitor oseltamivir leads to significant synergistic antiviral effect [60]. Moreover, pharmacokinetic studies suggested that compound 16 exhibited excellent metabolic stability and high oral availability [60]. Interestingly, our unpublished data have further revealed that compound 16 is orally active to protect mice from influenza H1N1 infection, and its synergy with oseltamivir was also observed.

#### 4.1.4. Others—Diverse Scaffolds toward Potent Inhibition against Group 1 HA Fusion

**Figure 6:**
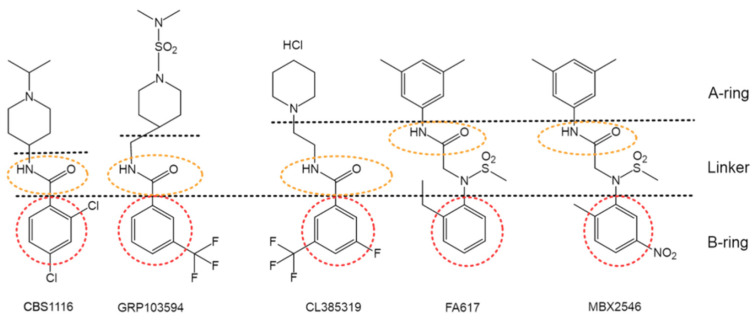
Group 1 specific influenza fusion inhibitors that share similar scaffold with CBS1116.

**Figure 7:**
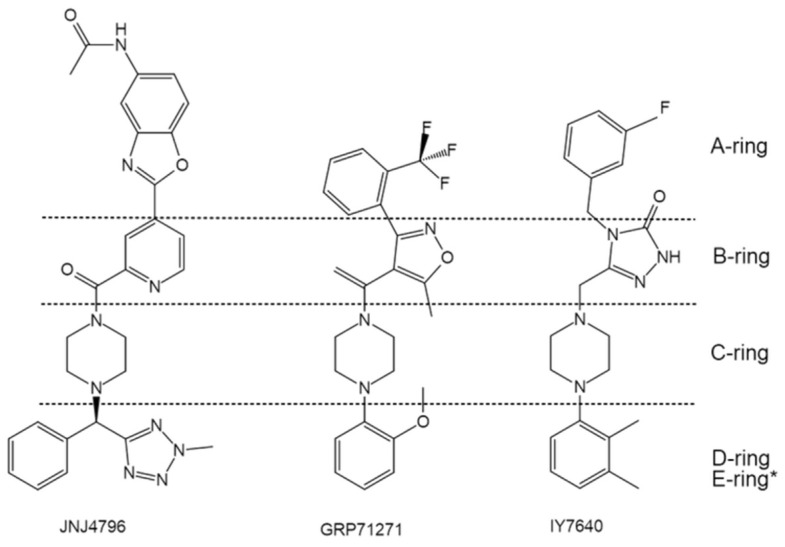
Comparison of the structures of GRP-71271 and IY7640 with JNJ4796. * JNJ4796 possesses an additional E-ring.

**Figure 8:**
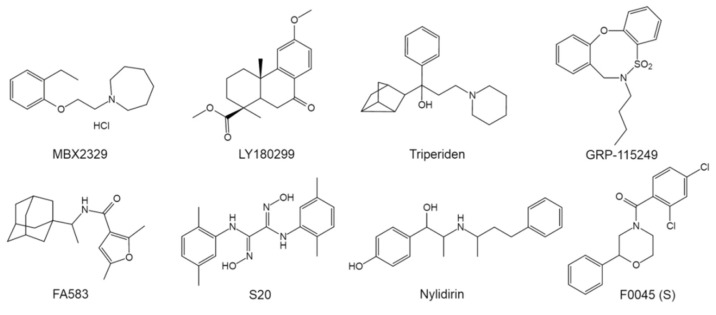
Structurally distinct inhibitors specifically targeting group 1 HA mediated fusion process.

In addition to the aforementioned lead structures, a large number of fusion inhibitors targeting group 1 HAs have been reported. Some of them share similar scaffolds. For example, CL-385319 [61,62], MBX2546 [63], FA617 [64], and GRP-103594 [65] share similar scaffold with BMY27709 and CBS1116 (A-ring/linker/B-ring), except for the linker, which might be elongated and the A-ring, which is variable (Figure 6). In addition, two JNJ4796-like inhibitors, GRP-71271 [65] and IY7640 [66], have also been reported (Figure 7). Moreover, there are several structurally distinct inhibitor classes, including MBX2329 [63], LY180299 and other diterpenoid derivatives [67,68,69], triperiden derivatives [68,70], GRP-115249 [65], FA583 [64], S20 [71], nylidrin [72], F0045(S) [73], and so on (Figure 8).

**Figure 9:**
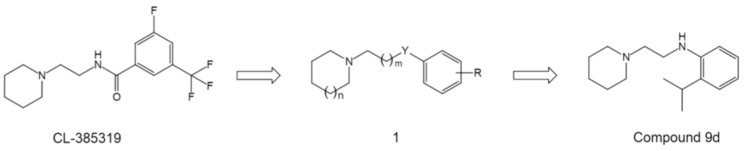
Design of general structure 1 using CL-385319 as starting point and the structure of compound 9d.

Overall, these structures provide either novel lead core skeletons as starting points for further optimization, or enrich our knowledge of SAR and direct novel strategies of chemical modification towards a more valuable drug candidate. For example, considering that the benzamide group of BMS-199945 could be replaced by an aniline group as in RO5464466 and RO5487624, Leiva et al. therefore reasoned that starting from the compound CL-385319, a series of aniline derivatives and related compounds of general structure **1** may also display anti-influenza activity (Figure 9) [74]. Inspired by this idea, the authors performed a comprehensive SAR study and generated a novel HA inhibitor compound 9d (Figure 9) [74].

### 4.2. Group 2 Specific Influenza Fusion Inhibitors

Compared to group 1 specific HA inhibitors, fewer group 2 specific ones have been identified. First, the models for group 2 IAV in vivo studies are less prevalent. For example, a robust mouse-adapted group 2 IAV strain is missing, in contrast to the useful H1N1/PR8 strain used for in vivo studies of group 1. Second, the intrinsic variations might have conferred less potent druggable pockets within group 2 than group 1 HAs. Nonetheless, it is clear that more attention should be paid to the discovery of novel group 2 specific HA inhibitors in the future.

#### 4.2.1. TBHQ—One of the Well-known Lead Molecules

**Figure 10:**
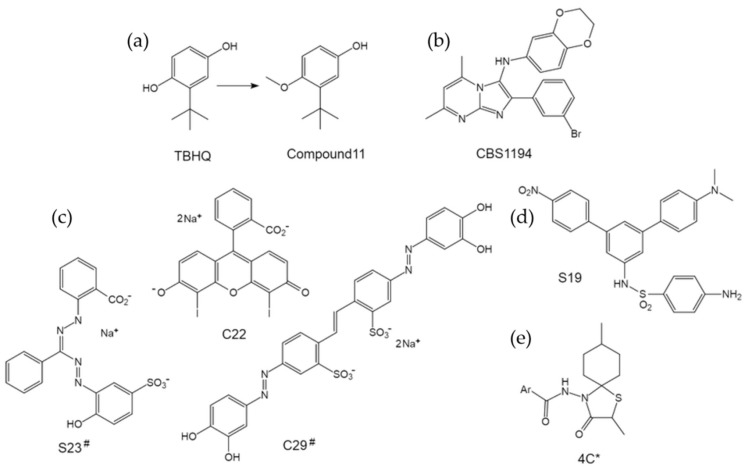
The structures of group 2 specific HA inhibitors targeting the fusion process. (**a**) TBHQ and its optimized derivative compound 11. (**b**) The structure of CBS1194. (**c**) Molecules that facilitate structural rearrangement of group 2 HAs. ^#^ the compounds did not show antiviral activities in bioassays. (**d**) The structure of S19. (**e**)The structure of compound 4c. * the inhibitory effect was defined within H3 subtype.

The small molecule tert-butylhydroquinone (TBHQ) has been well studied and shown to specifically inhibit group 2 IAVs by preventing HA-mediated fusion with low micromolar potency [75,76,77]. Although the relatively high anti-influenza activity in combination with the low toxicity makes TBHQ an attractive lead compound [78], its antioxidant property diminishes the enthusiasm due to the potential oxidation-reduction reactions or the covalent modification of host proteins. Interestingly, we and collaborators have clearly demonstrated that the anti-influenza activity of TBHQ was not due to its antioxidant property [79]. Moreover, by replacing the 1-hydroxyl group with a methoxy substituent to form an anisole, we obtained a TBHQ analogue, compound 11, yielding reduced chemical reactivity and about 10 times improvement of antiviral activity (Figure 10a) [79]. Our studies should have renewed interest in TBHQ analogues as influenza antivirals and can guide future efforts for chemical optimization.

#### 4.2.2. CBS1194—A Novel Scaffold That Deserves Further Optimization

As mentioned above, our group has been engaged in screening efforts for the discovery of influenza entry inhibitors of group 2 specificity in addition to group 1 specific ones. CBS1194 is one of the most potent hit compounds discovered to date (Figure 10b) [24]. Our data clearly demonstrated that CBS1194 broadly inhibits group 2 IAVs, and acts by preventing the HA rearrangement that is required for HA-mediated membrane fusion [24]. Using CBS1194 as the starting point, we have synthesized a series of derivative compounds that are ready for bioassays, in anticipation of more effective and valuable drug candidates.

#### 4.2.3. C22—A Facilitator of HA Conformational Change

All the group 1 and group 2 specific influenza fusion inhibitors described above act by preventing the low-pH induced conformational rearrangement of HA. Interestingly, Hoffman et al. previously identified a panel of compounds that facilitate rather than inhibit the HA conformational change, by destabilizing the HA at neutral pH [80]. Moreover, among these compounds, C22 (Figure 10c) can block HA-mediated membrane fusion and irreversibly decrease virus infectivity [80]. The discovery of C22 thus defines a new class of agents leading to anti-influenza drugs.

Beside C22, two other molecules that facilitate the conformational change have been identified as well, including C29 and S23 (Figure 10c) [80]. Unfortunately, C29 showed high cytotoxicity, while S23 exhibited no measurable effect on viral infectivity. However, further chemical optimizations can be used to fill these gaps between “in tube” activities and “in cellular” activities, e.g., by lowering the cytotoxicity of C29 without altering the HA-rearrangement facilitating activity. Both C29 and C23 may provide informative structures for development of potential antivirals in future [80].

#### 4.2.4. Others—A Long Way to Go

Along with C22, Hoffman et al. also identified compound S19 (Figure 10d), which prevents the conformational rearrangement of group 2 HAs and subsequently inhibit entry of group 2 IAVs [80]. Interestingly, in a quantitative assay for the HA conformational change, S19 showed to be less effective than TBHQ, however, in the infectivity assay, S19 exhibited much higher potency [80]. This discrepancy suggests a different mechanism of action of S19 than TBHQ, and it is interesting to further study the binding and action mode of S19 to HA, which may lead to a new class of group 2 HA inhibitors and guide drug design in the future.

In addition, a class of *N*-(1-thia-4-azaspiro [4.5]decan-4-yl)carboxamide inhibitors (e.g., 4c, Figure 10e) of influenza virus HA-mediated membrane fusion has been reported [81]. Subsequent SAR developed a most active analogue 5f, exhibiting an EC_50_ value of 1 nm against influenza A/H3N2 virus, and selectivity index of almost 2000 [82]. Although the spectrum of these inhibitors is narrow and defined within the H3 subtype, the structure basis alone or in complex with target HA should be instructive to achieve superior inhibitors of HA-mediated fusion.

### 4.3. Broad-Spectrum Influenza Fusion Inhibitors

An ideal HA-mediated fusion inhibitor should block the entry process of all subtypes of influenza viruses. This is hard to achieve since the structural features among subtypes of IAV and IBV vary a lot. Interestingly, Arbidol has been demonstrated to match the requirements as a pan-subtype influenza fusion inhibitor. Moreover, Arbidol has been administered for decades in Russia and China against influenza, with no major adverse effects reported [83]. It is noteworthy that the antiviral activity of Arbidol is not limited to influenza viruses, but Arbidol possesses vast potential as a broad-spectrum antiviral agent against diverse enveloped and non-enveloped viruses including hepatitis B virus, hepatitis C virus, chikungunya virus, reovirus, hantaan virus, ebola virus, coxsackie virus B5 and the emerging severe acute respiratory syndrome coronavirus 2 [84,85,86]. Multiple studies have suggested that Arbidol is a cell-targeting antiviral which can incorporate into cellular membranes, modify their physico-chemical properties, and subsequently block virus entry [84]. However, crystal structures of Arbidol in complex with group 2 HAs indicated that Arbidol can bind in a hydrophobic cavity in the HA trimer stem at the interface between two protomers, suggesting a different mechanism of action [87].

**Figure 11:**
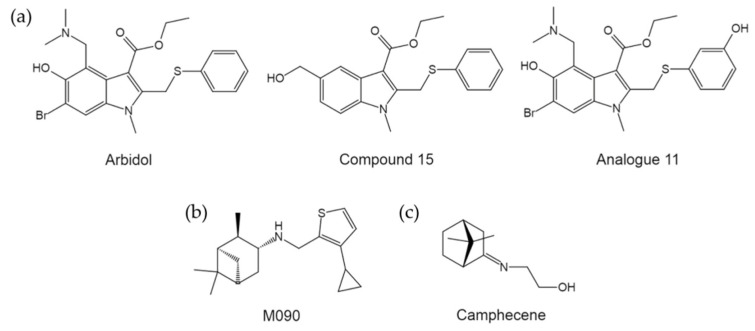
The structures of pan-subtype HA fusion inhibitors. (**a**) The structures of Arbidol and its derivatives. (**b**) The structure of M090. (**c**) The structure of camphecene.

Previously, Brancato et al. designed and developed two series of indole derivatives structurally related to Arbidol and the antiviral activities were probed. As a result, compound 15 (Figure 11a) was identified to be more potent than Arbidol against certain subtypes of influenza A viruses [88]. Particularly, compound 15 exhibited a much greater affinity and preference for binding group 2 than group 1 HAs, in contrast to Arbidol [88]. More recently, Wright et al. noticed that there is underutilized space in the binding pocket for Arbidol within H3 and H7 HAs based on HA-arbidol co-crystal structures resolved by Kadam et al. [87]. Using rational design, the authors then generated a series of Arbidol analogues, resulting in analogue 11 with impressive increases in affinity for both group 1 and group 2 HAs (Figure 11a) [89].

Of particular note, during the course of developing inhibitors targeting the IAV M2 proton channel carrying the amantadine-resistant S31N mutation (AM2-S31N), Zhao et al. identified M090 (Figure 11b), an analogue of pinanamine-based M2 inhibitors, to inhibit viral replication through AM2-S31N independent mechanisms instead of AM2-S31N blockage [90]. Subsequent drug resistance selection identified several mutations in HA, in combination with molecular dynamics simulations and hemolytic fusion inhibition studies, these data strongly suggested that M090 acts by targeting HA mediated fusion [90]. Moreover, M090 exhibited inhibition against a broad spectrum of IAVs, including both group 1 and group 2 subtypes, with EC_50_ values ranging from 0.1 to 10 μm [90]. In addition, Zarubaev et al. identified an imino-derivative of camphor, termed camphecene (Figure 11c), as a novel fusion inhibitor targeting HAs of broad range [91]. Similar to M090, the camphor-based cage compounds were initially designed and synthesized to block amantadine-and rimantadine-resistant M2 channels [92]. These results are encouraging for further development of potential pan-subtype influenza inhibitors targeting the fusion machinery.

## 5. Structure-Based Perspectives

A major contribution to the advances in the development of HA inhibitor comes from structural biology, in particular from the many studies on influenza HA alone or in complex with potent inhibitors [93]. Although it is interesting to explore conserved druggable pockets besides RBD site in the plastic head domain in future, at present the fusion machinery of HA is a more preferred target for the discovery and design of novel small molecule inhibitors. Previously, the crystal structures of influenza HAs have demonstrated the group-specific basis in regions that are prominent in the rearrangements required for membrane fusion [94,95]. The fact that most of the influenza fusion inhibitors work in a group-specific manner suggests that they may bind in one of these regions, and our understanding on the binding mode of fusion inhibitors can guide future efforts to further optimize this class of compounds. Moreover, the structural basis for fusion inhibition may lead to rational design of more ideal broad-spectrum fusion inhibitors against all subtypes of IAV and IBV.

So far to our knowledge, the binding properties of five small molecule fusion inhibitors have been unraveled, based on the co-crystal structure of HA in complex with the individual molecules. Among the five molecules, JNJ4796, CBS1117 (an analogue of CBS1116) and F0045(S) represent group 1 specific fusion inhibitors, while TBHQ and Arbidol represent group 2 specific ones. Note that although Arbidol can also inhibit the fusion mediated by group 1 HAs, the structure of group 1 HA in complex with Arbidol has not been resolved.

**Figure 12:**
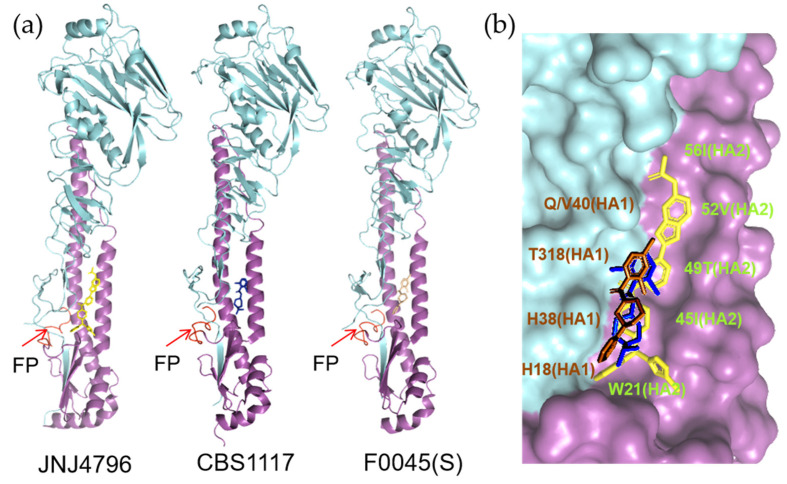
Binding mode of group 1 specific HA inhibitors JNJ4796, CBS1117 and F0045(S). (**a**)Crystal structures of group 1 HA in complex with molecules JNJ4796 (PDB code: 6cfg), CBS1117 (PDB code: 6vmz) and F0045(S) (PDB code: 6wcr). The fusion peptides (FPs) are shown in red and indicated by arrows. (**b**) Alignment of bound complexes with JNJ4796 (yellow), CBS1117 (blue) and F0045(S) (brown). In the surface representation of H5 HA, the HA1 and HA2 subunits are colored cyan and pink, respectively. The critical interacting residues were indicated.

Interestingly, the binding sites of JNJ4796, CBS1117 and F0045(S) overlap significantly, localized in a pocket near the fusion peptide (Figure 12a) [48,73,96]. JNJ4796 is the biggest compound, containing five rings occupying a large region of the target pocket, nonetheless, the binding mode of CBS1117 and F0045(S) are strikingly similar to the binding modes of the B, C, and D rings of JNJ4796 (Figure 12b) [48,73,96]. Notably, the B-ring of CBS1117 reacts with HA similarly to both the C- and D-ring of JNJ4796 [96]. It will be of great interest to elaborate the CBS1117 and F0045(S) molecules into nearby unoccupied regions at the binding site to improve the binding and neutralization activity against influenza viruses.

**Figure 13:**
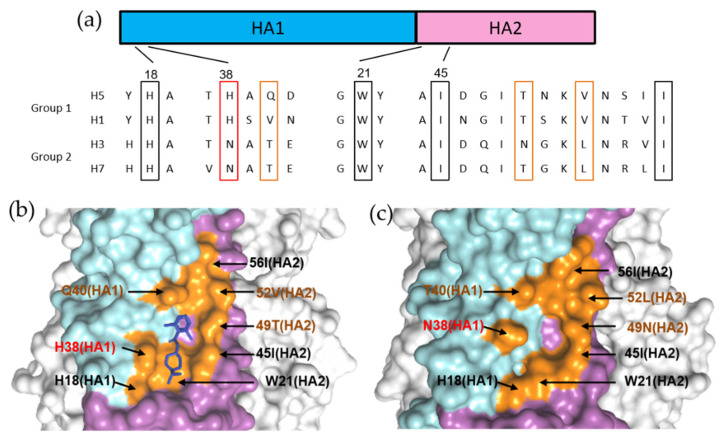
Group 1specific binding of compoundCBS1117. (**a**) Sequence alignment between group 1 (H1 and H5) and group 2 (H3 and H7) HAs. (**b**) Structure of H5 HA in complex with CBS1117 (blue). (**c**) Structure of H3 HA (PDB code: 5t6b). The essential residues involved in CBS1117 binding site are shown in orange. The HA2 position 38 is indicated in red, while other critical variable residues are indicated in brown.

It is speculated that binding of these molecules can stabilize the pre-fusion conformation of HA by blocking the release of fusion peptide. Furthermore, the group 1 specificities of the three inhibitors were rationalized and primarily directed to HA1 position 38, where a group 1 conserved key residue HA1–H38 is substituted to a conserved glycosylated N38 within group 2 HAs (Figure 13a) [48,73,96]. The glycosyl group induced steric hindrance, which is argued to be the most important factor that renders group specificity (Figure 13b,c). Besides, the orientations of pan-conserved residues HA1–H18 and HA2–W21, and variable residues such as HA2 positions 49 and 52 in the binding site may also lead to group specificity (Figure 13b,c) [48,73,96]. It is important to take these distinct structural features into consideration for the future design of group 2 specific or broad-spectrum inhibitors targeting the corresponding druggable pocket.

**Figure 14:**
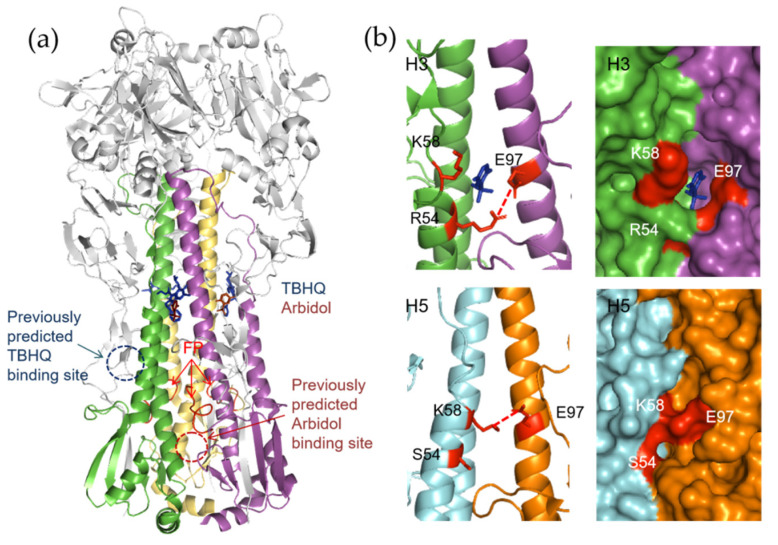
Binding modes of TBHQ and Arbidol to group 2 HA. (**a**) The crystal structure of Arbidol/TBHQ in complex with H3 HA is represented with the HA1 shown in gray. The protomers of HA2 trimer is shown in blue, pink, and yellow cartoon separately, while TBHQ and Arbidol are shown as blueand brown sticks, respectively. The N-terminal fusion peptides (FPs) in the trimer has been highlighted in red and indicated by red arrows. The previously predicted binding sites for TBHQ and Arbidol from docking studies are marked by blue and red dashed circles, respectively. (**b**) The structural basis for HA group-specific inhibition by TBHQ. Comparison of the TBHQ binding site of H3 and corresponding position of H5 clearly shows that the extra turn of the short α-helix in the group 1 HAs precludes TBHQ binding. Potential hydrogen bonds are shown as dotted lines.

In contrast, both TBHQ and Arbidol bind in a hydrophobic cavity in the HA trimer stem loop at the interface between two protomers (Figure 14a) [76,87]. It is noteworthy that previously TBHQ and Arbidol were predicted by in silico studies to bind at distinct pockets close to the fusion peptide, which differ from their actual binding sites (Figure 14a) [76,87,97]. Moreover, the resistance mutations to TBHQ or Arbidol usually do not map around the compound binding site, which contradicts the common notion that escaped mutations map directly to the site of action [76,87]. Instead, these resistance mutations mainly involve residues that interact with the fusion peptide, suggesting a mode of action by destabilizing the neutral form of HA and increasing the pH of membrane fusion [76,87,97].

The structural differences of group 1 and group 2 HAs in the vicinity of the TBHQ binding site could account for the group-specific activity of the compound. As shown in Figure 14b, the substitution of group 2 conserved residue HA2 R54 to group 1 conserved S54 leads to the diminish of an intermonomer salt bridge between R54 and E97. Alternatively, the E97 forms a less optimal salt bridge with the residue HA2 K58, which is conserved in all HAs. As a result, an extra turn is present at the C terminus of the short α-helix (residues 56–58), and the access of TBHQ to group 1 HAs is apparently blocked (Figure 14b). When it comes to Arbidol, it is unlikely that the compound binds to group 1 HAs at the same site identified in group 2 HAs, but Arbidol might occupy a distinct group 1specific binding site [87]. Further resolution of the crystal structure of a group 1 HA in complex with Arbidol may not only improve our understanding of the compound’s pan-subtype mode of action, but also guide the future optimization of Arbidol and the design of novel broad-spectrum HA fusion inhibitors.

For other HA fusion inhibitors, the co-crystallization structures in complex with target HAs have not been resolved, and their actual binding modes and mechanisms of action remain obscure. Nonetheless, in silico studies with these compounds have predicted several valuable potent binding pockets, including group 1 and group 2 specific ones, as well as cavities that are conserved among all subtypes. For example, compound MBX2546 was predicted to bind at the center within the stem of HA trimer [98]. In contrast to the fact that there are three binding sites per HA trimer for JNJ4796, CBS1117, F0045(S), TBHQ and Arbidol, only one molecule MBX2546 is modeled to bind to HA trimer [98]. Moreover, using WaterLOGSY (Water Ligand Observed via Gradient Spectroscopy) NMR we and our collaborators have demonstrated that MBX2546 can bind to both H5 (group 1 HA) and H3 (group 2 HA), which contradicts with the group 1 specific antiviral activity. Therefore, it was supposed that specific binding of MBX2546 to HA could not guarantee efficient fusion inhibition, while specific interactions between HA and MBX2546 to stabilize the non-fusing conformation of HA trimer is also required [98]. This phenomenon is not unique, since BMY27709 analogues BMS-198254 and BMS-195161 also bind to HA specifically, while they are inactive to block conformational changes of HA but act as antagonists of the inhibitory effects of BMY27709 [43]. It is of great interest to further study the individual structure of H5 and H3 in complex with MBX2546 to identify stabilizing interactions between MBX2546 and H3 or both H5 and H3, which may serve as a guide for the generation of novel group 2 specific or pan-subtype fusion inhibitors.

The potent binding site of broad-spectrum HA inhibitor M090 has also been predicted. M090 occupies a pocket located at the interface of the long α-helical segment and a loop of the HA2 monomer, thus inhibiting virus-mediated membrane fusion by “locking” the bending state of HA2 during the conformational rearrangement process [90]. Consistent with the observed pan-subtype inhibition against influenza virus infection, sequence alignment suggests this binding region is conserved and could be used to design advanced inhibitors that target both group 1 and group 2 of HAs [90]. If this novel pocket can be validated, it will become one of the most interesting targets to explore potentially broad-spectrum anti-influenza drugs in the future.

## 6. Conclusions

It is of great concern that resistant strains against currently available anti-influenza drugs are frequently identified in human or avian influenza virus [99]. Driven by structural and functional characterization of influenza HA, the development of potent HA inhibitors has raised hopes for new antiviral therapies. As the most potent HA-targeted drug candidates are directed to the fusion machinery and are group specific, it is of great interest to develop group 1 and group 2 specific anti-influenza drugs separately and to use the appropriate inhibitor for each influenza outbreak. Further, the discovery, chemical modification, and MOA studies of pan-subtype HA fusion inhibitors may lead to more valuable broad-spectrum anti-influenza drugs targeting both group 1 and group 2 HAs. Moreover, since the combination of virus specific compounds can increase efficacy and decrease the incidence of viral resistance, similar to HAART for treatment of HIV infections, it is possible that two subtype specific fusion inhibitors or a broad-spectrum one could be paired with a drug targeting a different stage of the viral life cycle, such as oseltamivir or baloxivir, to produce a more effective cocktail therapy.

## Data Availability

Not applicable.

## Abbreviations

IAV, influenza A virus; NA, neuraminidase; HA, Hemagglutinin; HEF, haemagglutinin-esterase-fusion; SA, sialic acid; RBS, receptor binding site; PT, pentacyclic triterpenoid; OA, oleanolic acid; MOA, Mechanism of action; SAR, structure-activity relationship; bnAb, broadly neutralizing antibody; FP, fusion peptide; AlphaLISA, amplified luminescentproximity homogeneous assay; HTS, high-throughput screening; TBHQ, tert-butylhydroquinone; WaterLOGSY, Water Ligand Observed via Gradient Spectroscopy.

## Appendix A

**Table A1:** The small molecule HA fusion inhibitors and their group specificities, in vitro antiviral activities and in vivo efficacies.

Compound	Group Specificity	In Vitro Activity EC_50_ (μM) ^a^/CC_50_ (μM) ^b^	In Vivo Effective	Ref.
RO5487624	1	0.09–0.45/71	Yes	[46,47]
JNJ4796	1	0.012–3.24/- ^c^	Yes	[48]
CBS1116	1	0.4/160	-	[21]
compound 16	1	0.072/>100	Yes ^d^	[60]
GRP-103594	1	0.45/>100	-	[65]
CL-385319	1	0.38–2.66/47.5	-	[61,62]
9d	1	1.7–4.6/>100	-	[75]
FA-617	1	0.007–0.087/-	-	[64]
MBX2546	1	0.3–5.8/>100	-	[63]
GRP-71271	1	0.28/>100	-	[65]
IY-7640	1 (H1N1)	0.07–7.1/-	Yes	[66]
MBX2329	1	0.29–0.53/>100	-	[63]
LY180299	1	0.03–0.1/>66	-	[67]
Triperiden	1	-/>100	-	[70,71]
GRP-115249	1	0.33/>100	-	[65]
FA-583	1	0.044–0.080/-	-	[64]
S20	1	0.08–0.15/40	-	[72]
Nylidirin	1	1.7–3.5/549.2	Yes	[73]
F0045(S)	1	0.5–16.2/>157	-	[74]
TBHQ	2	6/>50	-	[76,77,78]
TBHQ derived compound 11	2	0.6/>50	-	[80]
CBS1194	2	0.36–3.7/>100	-	[24]
C22	2	8.0/1000	-	[81]
S19	2	0.8/>500	-	[81]
4c	2 (H3N2)	3.3–23/>100	-	[82]
5f	2 (H3N2)	0.001/1.5	-	[83]
Arbidol	Broad spectrum	8.4–62.8/-	Approved in Russia and China	[84,88]
M090	Broad spectrum	0.10–6.85/25	-	[91]
camphecene	Broad spectrum	3.6–83.8/701	Yes	[92]

^a^ EC_50_, concentration required to produce 50% of a maximal effect. Of note, the EC_50_ values were obtained from different antiviral assays against diverse influenza strains. These data are for reference only and it makes no sense to compare with each other; ^b^ CC_50_, 50% cytotoxic concentration; ^c^ data not available; ^d^ Unpublished data.
